# Frequent exacerbators in severe asthma: Focus on clinical and transcriptional factors

**DOI:** 10.1002/ctm2.860

**Published:** 2022-05-11

**Authors:** Neil C. Thomson

**Affiliations:** ^1^ Institute of Infection Immunity & Inflammation University of Glasgow Glasgow Scotland UK

1

Approximately 5% of adults with asthma have severe disease. Despite its relatively low prevalence, patients with severe asthma experience substantial morbidity due to poorly controlled symptoms, exacerbations, multiple comorbidities, and the adverse effects of systemic corticosteroids. These patients generate high healthcare costs from prescribed medications, outpatient care, and hospital admissions due to exacerbations. Surveys of adults with severe asthma have shown that most experience one or more exacerbations each year despite recommended therapies. The term “frequent exacerbator” is used to describe a clinical phenotype with ≥ 2^1^, ^2^ or ≥ 3^1^, ^3^ severe exacerbations in the previous year. Risk factors for frequent exacerbations include a history of severe exacerbations, chronic airflow obstruction, raised biomarkers of type 2 eosinophilic airway inflammation, a history of cigarette smoking, a high body mass index, chronic sinusitis, gastroesophageal reflux disease, and low socioeconomic status.^4^ Management of frequent exacerbations in severe asthma includes the identification and targeting of treatable traits^5^ and the implementation of GINA recommendations on the treatment of severe asthma.^6^ Despite progress in understanding the clinical features, inflammatory pathways, and therapies available to prevent exacerbations in severe asthma,^4^ data are limited on the best approach to identify individuals who are at greatest risk of frequent exacerbations and on the inflammatory mechanisms underlying frequent exacerbator status.

In the current issue of *Clinical and Translational Medicine*, Hoda and colleagues^7^ reported the results of a study designed to provide insights into the clinical and immunological factors underlying frequent exacerbations in 420 adults with severe asthma recruited to the U‐BIOPRED (the Unbiased Biomarkers for the Prediction of Respiratory Disease Outcome) cohort. Baseline clinical variables and transcriptional data in blood, bronchial and nasal epithelial brushings, bronchial biopsies, and sputum cells among frequent exacerbators, defined as ≥ 2 exacerbations in the previous year, were compared with infrequent exacerbators, defined as a maximum of one exacerbation in the previous year. Longitudinal data were also analysed from 317 individuals with persistent frequent exacerbations or persistent infrequent exacerbations during 1 year of follow‐up.

The finding that two‐thirds of the U‐BIOPRED cohort were frequent exacerbators (≥ 2) confirms previous reports of a high prevalence of frequent exacerbators in severe asthma,^1^, ^2^ including data from the UK Severe Asthma Registry, which found that 71% of adults were frequent exacerbators (≥ 3).^3^ Collectively, these studies demonstrate the clinical importance of severe exacerbations among patients with severe asthma. Interestingly, longitudinal data showed that one‐third of frequent exacerbators and one‐third of infrequent exacerbators reversed exacerbation status during 1 year of follow‐up, suggesting that the exacerbator phenotype in severe asthma can change over time.

The study highlights several clinical variables associated with frequent exacerbations, including greater daily short‐acting beta_2_‐agonist (SABA) use and a high asthma control questionnaire (ACQ)‐5 score. In a large UK severe asthma registry population of 1592 patients, a high ACQ score (> 1.5) had the strongest association with frequent exacerbations irrespective of maintenance oral corticosteroid status.^3^ Whether a poor ACQ score alone or as a component of a composite risk score is useful in identifying frequent exacerbators among adults with severe asthma in clinical practice will require prospective studies. In the U‐BIOPRED study, former smoking was associated with increased exacerbations at baseline,^7^ whereas in the UK Severe Asthma Registry population, a past smoking history correlated with frequent exacerbations only in patients on maintenance oral corticosteroids.^3^ Unexpectedly, Hoda and colleagues^7^ found that current smoking status was associated with a reduced risk of persistent frequent exacerbations, whereas other studies have reported that current smoking status^8^ or high pack‐year history^1^ was associated with an increased risk of exacerbations in severe asthma. The reason for the contrasting findings is uncertain but may be due to differences between study populations in the proportion of current and former smokers, cumulative smoking history, oral corticosteroid use, or other variables.

Over 100 gene signatures that might be involved in frequent exacerbations among adults with asthma were examined in the different biological samples collected by Hoda and colleagues.^7^ CEA Cell Adhesion Molecule 5 (CEACAM5) expression in bronchial biopsies was increased in frequent exacerbators compared to infrequent exacerbators, whereas transcripts in other compartments did not differ between exacerbator subgroups. Could CEACAM5 be implicated in the pathogenesis of frequent exacerbations? The CEACAM immunoglobulin superfamily is involved in numerous cell functions including differentiation, proliferation, and apoptosis.^9^ In adults with severe asthma, bronchial epithelial CEACAM5 expression and gene signatures typical of the inflammatory airway response driven by bacterial infection are upregulated compared to healthy controls.^10^ CEACAM receptors, including CEACAM5, have been implicated in host defense against bacterial infections, particularly *Moraxella catarrhalis* and nontypable *Haemophilus influenzae*.^9^ The binding of bacteria to CEACAMs is thought to mediate bacterial adhesion and contribute to colonisation of the airways. Hoda and colleagues^7^ speculate that CEACAM5 could be involved in exacerbations due to bacterial infections among frequent exacerbators, possibly after viral respiratory infections.^9^ Confirmation of these findings could lead to the development of therapies targeting the epithelial CEACAM5 receptor among frequent exacerbators. Additionally, the frequent exacerbator status was associated with increased expression of type 1 inflammatory (virus‐induced Th1 response), type 2 inflammatory, and corticosteroid insensitivity pathways in nasal brushings, bronchial biopsies, or sputum cells. There are limitations associated with these data, including a low number of transcription samples for some compartments, such as bronchial biopsies (13% of patients) and bronchial brushings (16% of patients), and the increase in CEACAM5 expression among frequent exacerbators at the baseline assessment was not confirmed in the 1‐year follow‐up study, possibly due to a reduced sample size of persistent frequent exacerbators.

In conclusion, the study by Hoda and colleagues^7^ has provided new data on transcriptional factors in blood and airway samples associated with frequent exacerbations in severe asthma. Interestingly, increased CEACAM5 expression in bronchial biopsies was associated with frequent exacerbator status, whereas transcripts in other compartments did not differ between exacerbator subgroups. The findings need to be replicated in other populations of frequent exacerbators with severe asthma. Establishing whether the frequent exacerbating phenotype is associated with specific inflammatory pathways may contribute to the development of novel targeted therapies.

## CONFLICT OF INTEREST

No conflict of interest.
